# Sex difference in the discordance between Abbott Architect and EuroImmun serological assays

**DOI:** 10.7717/peerj.15247

**Published:** 2023-07-17

**Authors:** Joel D. Hartsell, Daniel T. Leung, Yue Zhang, Julio C. Delgado, Stephen C. Alder, Matthew H. Samore

**Affiliations:** 1 Department of Population Health, University of Utah, Salt Lake City, UT, United States of America; 2 Epi-Vant LLC, Salt Lake City, UT, United States of America; 3 Department of Pathology, University of Utah, Salt Lake City, UT, United States of America; 4 Department of Internal Medicine, University of Utah, Salt Lake City, UT, United States of America; 5 ARUP Laboratories, Salt Lake City, UT, United States of America; 6 Department of Entrepreneurship and Strategy, University of Utah, Salt Lake City, UT, United States of America; 7 Veteran Affairs, Salt Lake City, UT, United States of America

**Keywords:** COVID-19, Serology, Diagnostic screening programs, Discordant results, Positive predictive value, Sex driven discordance

## Abstract

**Background:**

This study evaluated the discordance between Abbott Architect SARS-CoV-2 IgG and EUROIMMUN SARS-COV-2 ELISA in a seroprevalence study.

**Methods:**

From June 10 to August 15, 2020, 8,246 specimens were dually evaluated by the Abbott Architect SARS-CoV-2 IgG (Abbott) and the EUROIMMUN SARS-CoV-2 ELISA (EI) assays. Sex-stratified phi correlation coefficients were calculated to evaluate the concordance between Abbott and EI assay’s quantitative results. Multivariable mixed-effect logistic models were implemented to evaluate the association between assay positivity and sex on a low prevalence sample while controlling for age, race, ethnicity, diabetes, cardiovascular disease, hypertension, immunosuppressive therapy, and autoimmune disease.

**Results:**

EI positivity among males was 2.1-fold that of females; however, no significant differences in Abbott positivity were observed between sexes. At the manufacturer-recommended threshold, the phi correlation coefficient for the Abbott and EI qualitative results among females (Φ = 0.47) was 34% greater than males (Φ = 0.35). The unadjusted and fully adjusted models yielded a strong association between sex and positive EI result for the low prevalence subgroup (unadjusted OR: 2.24, CI: 1.63, 3.11, adjusted OR: 3.40, CI: 2.15, 5.39). A similar analysis of Abbott positivity in the low prevalence subgroup did not find an association with any of the covariates examined. Significant quantitative and qualitative discordance was observed between Abbott and EI throughout the seroprevalence study. Our results suggest the presence of sex-associated specificity limitations with the EI assay. As these findings may extend to other anti-S assays utilized for SARS-CoV-2 seroprevalence investigations, further investigation is needed to evaluate the generalizability of these findings.

## Introduction

In response to the emergence of sudden acute respiratory syndrome coronavirus 2 (SARS-CoV-2), the urgent need for assessment of population prevalence led to the release of serological assays with abbreviated validation under the Food and Drug Association’s Emergency Use Authorization (EUA) (Ainsworth et al., 2020; Karpe et al., 2018; Rikhtegaran Tehrani et al., 2020). While the initial results demonstrated high sensitivity and specificity, studies have found evidence suggestive that the clinical performance for some is much lower than initially anticipated (Prince et al., 2020; Tang et al., 2020). As the Abbott Architect SARS-CoV-2 immunoglobulin G (IgG) (Abbott) and EUROIMMUN SARS-COV-2 enzyme-linked immunoassay (ELISA) (EI) have often been treated as a comparable diagnostic in seroprevalence surveys, understanding their respective limitations is critical to sero-epidemiological investigations (Prince et al., 2020; Tang et al., 2020).

The Utah Health and Economic Recovery (HERO) Project is a seroprevalence survey aimed to improve the understanding of community-based SARS-CoV-2 activity. As a part of HERO, a sample of Utah residents took part in diagnostic screening and completed a survey assessing demographic and epidemiologic factors. Serum from most participants were dually evaluated by both Abbott and EI at the same encounter, thus the data collected throughout this project provides a unique opportunity to investigate their real-world performance and the factors contributing to underlying discordance.

While many studies have described sex-dependent differences in immune response and severity of coronavirus disease 2019 (COVID-19), no studies have previously described sex-associated discordance between immunoassays (Agrawal et al., 2021; Bunders & Altfeld, 2020; Grzelak et al., 2021; Haitao et al., 2020; Lakbar et al., 2020; Maleki Dana et al., 2020; Muecksch et al., 2021; Takahashi et al., 2020). This study evaluated discordance between Abbott Architect SARS-CoV-2 IgG and EUROIMMUN SARS-COV-2 ELISA in a real-world setting.

## Materials & Methods

From June 10 to August 15, 2020, a total of 8,246 specimens were evaluated by both the Abbott Architect SARS-CoV-2 IgG (Abbott) and the EUROIMMUN SARS-CoV-2 ELISA (EI) assays as part of the HERO Project. The Abbott assay detects anti-SARS-CoV-2 IgG against the nucleocapsid protein (anti-N), and the EI assay detects anti-SARS-CoV-2 IgG directed against the S1 domain of viral spike protein (anti-S1) (Rychert et al., 2021; Tang et al., 2020). Of those screened by both diagnostic tests, 99% (*n* = 8,166) completed the survey assessing demographic and epidemiologic factors.

The University of Utah Institutional Review Board determined the HERO project to be non-research public health surveillance and waived the requirement for documented consent. The review board determined that use of these data for analysis to understand the dynamics of SARS-CoV-2 was exempt from further review (IRB_00132598).

Summary statistics stratified by Abbott and EI assay results were calculated for sex, age, race, ethnicity, diabetes, cardiovascular disease, hypertension, immunosuppressive therapy, autoimmune disease, self-reported previous positive polymerase chain reaction (PCR), positive PCR at the time of screening, known exposure to COVID-19 case, and history of anosmia or ageusia. Fisher’s exact tests were performed to evaluate significant association between assay results and demographic and epidemiological factors.

Sex-stratified phi correlation coefficients were calculated to quantify the degree of concordance between Abbott and EI assay’s quantitative results for the total sample population. Concordance was evaluated at the manufacturer-recommended and adjusted cut-points for Abbott (quantitative threshold: 1.0 to 1.8) and EI (quantitative threshold: 0.7−2.0). The manufacturer recommended cut-points for positivity were defined as 1.1 for EI and 1.4 for Abbott.

Participants who self-reported a previous positive PCR, had a positive PCR at the time of screening, claimed anosmia or ageusia after March 1, 2020, or claimed an exposure with a known case were classified as the high prevalence subgroup. These factors were utilized as they are potentially predictive of true positivity. The remaining participants were categorized in the low prevalence subgroup. While we did not know which of the discordant results was a true positive or false positive, by stratifying the results into these two subgroups we generated a group with a greater proportion of samples of false positives. This approach enabled the assumption that the positive predictive value was lower in the low prevalence subgroup; therefore, a greater proportion of people who tested positive were false positives. Sex- and subgroup-stratified summary statistics for the low prevalence subgroup were calculated and reported below. Differences between stratum were evaluated utilizing Fisher’s exact test.

Multivariable mixed-effect logistic models were implemented to evaluate the association between assay positivity and sex in the low prevalence subgroup while controlling for age, race, ethnicity, diabetes, cardiovascular disease, hypertension, immunosuppressive therapy, and autoimmune disease (Akama-Garren & Li, 2021; Chen et al., 2021a; Fox et al., 2020; Mouliou & Gourgoulianis, 2021; Nishiga et al., 2020). This analysis was limited to the variables captured in a self-reported survey as a part of the HERO Project. These covariates were selected due to their association with severity of illness, immune response, or known differences in the prevalence of COVID-19 in Utah (Akama-Garren & Li, 2021; Chen et al., 2021a; Fox et al., 2020; Mouliou & Gourgoulianis, 2021; Nishiga et al., 2020). Household ID was included in the model as a random intercept to account for clustering. Authors considered the use of a fixed effect and isotonic regression for this analysis, but felt a mixed-effect model was better suited to account for variability within and across participants as multiple participants from the same household may be captured during the study period. Odds ratios and 95% confidence intervals were evaluated. The variance inflation factor was evaluated to assess for multicollinearity using the performance v0.10.1 package.

Sensitivity analyses were performed to evaluate the impact of alternate cut points that yielded the greatest reduction without reducing the positive concordance in the low prevalence subgroups.

Statistical analyses were performed with R v4.0.3 (R Core Team, 2020). The mixed effect logistic regression was implemented with the lme4 v1.1-27 package (https://cran.r-project.org/web/packages/lme4/index.html).

## Results

### Total population sample

Significant differences in positivity were observed between EI (3.0% positivity) and Abbott (1.4% positivity). Factors that reflected exposure or recent infection (*i.e.,* previous positive PCR, positive PCR at the time of screening, known exposure, or recent history of anosmia or ageusia) were strongly associated with positivity for both assays. Additionally, hypertension and ethnicity were associated with Abbott and EI positivity. EI positivity among males was 2.1-fold that of females; however, no significant differences in Abbott positivity were observed between sexes. While race was associated with Abbott positivity in the crude analysis, no association was observed between EI positivity and race. Age, diabetes, cardiovascular disease, immunosuppressive therapy, and autoimmune disease were not associated with Abbott or EI positivity (Table 1).

**Table 1:** Demographic and epidemiologic factors by Abbott and EI result. The table shows the demographic and epidemiologic factors by assay result. EI and Abbott results are displayed at manufacturer recommended thresholds, 1.1 and 1.4, respectively. Fisher’s exact test was used to evaluate null and alternate hypothesis. H_0_: test result and parameters are independent. H_1_: test result and parameters are not independent.

** **	** **	**Abbott result**			**EuroImmun result**					
** **	**Level**	**Negative**	**Positive**	*p*		**Negative**	**Positive**		*p*	
N		8,053	113			7,919	247			
Sex (%)	Female	4302 (53.4)	63 (55.8)	0.69		4269 (53.9)	96 (38.9)		<0.001	
	Male	3751 (46.6)	50 (44.2)			3650 (46.1)	151 (61.1)			
Age (mean (SD))		44.8 (18.9)	44.2 (17.0)	0.743		44.9 (18.9)	41.9 (18.2)		0.015	
							
Race (%)	American Indian or Alaska Native	44 (0.5)	1 (0.9)	0.005		43 (0.5)	2 (0.8)		0.348	
	Asian	193 (2.4)	1 (0.9)			192 (2.4)	2 (0.8)		
	Black	37 (0.5)	1 (0.9)			38 (0.5)	0 (0.0)			
	Multi-racial	173 (2.1)	5 (4.4)			170 (2.1)	8 (3.2)		
	Native Hawaiian or Other Pacific Islander	30 (0.4)	0 (0.0)			29 (0.4)	1 (0.4)			
	Unknown	197 (2.4)	9 (8.0)			197 (2.5)	9 (3.6)		
	White	7379 (91.6)	96 (85.0)			7250 (91.6)	225 (91.1)			
Ethnicity (%)	Hispanic	698 (8.7)	30 (26.5)	<0.001		693 (8.8)	35 (14.2)		0.005	
	Non-Hispanic	7355 (91.3)	83 (73.5)			7226 (91.2)	212 (85.8)			
Diabetes (%)	No	7529 (93.5)	109 (96.5)	0.28		7402 (93.5)	236 (95.5)		0.24	
	Yes	524 (6.5)	4 (3.5)			517 (6.5)	11 (4.5)			
Cardiovascular Disease (%)	No	7714 (95.8)	109 (96.5)	0.907		7587 (95.8)	236 (95.5)		0.968	
	Yes	339 (4.2)	4 (3.5)			332 (4.2)	11 (4.5)			
Hypertension (%)	No	6983 (86.7)	106 (93.8)	0.038		6862 (86.7)	227 (91.9)		0.021	
	Yes	1070 (13.3)	7 (6.2)			1057 (13.3)	20 (8.1)			
Immunosuppressive Therapy (%)	No	7974 (99.0)	112 (99.1)	1		7840 (99.0)	246 (99.6)		0.546	
	Yes	79 (1.0)	1 (0.9)			79 (1.0)	1 (0.4)			
Autoimmune Disease (%)	No	7635 (94.8)	105 (92.9)	0.494		7510 (94.8)	230 (93.1)		0.294	
	Yes	418 (5.2)	8 (7.1)			409 (5.2)	17 (6.9)			
Previous Positive (%)	No	8043 (99.9)	77 (68.1)	<0.001		7909 (99.9)	211 (85.4)		<0.001	
	Yes	10 (0.1)	36 (31.9)			10 (0.1)	36 (14.6)			
Positive PCR (%)	Not Detected	8044 (99.9)	92 (81.4)	<0.001		9 (0.1)	21 (8.5)		<0.001	
	Detected	9 (0.1)	21 (18.6)			7910 (99.9)	226 (91.5)			
Anosmia or Ageusia (%)	No	7963 (98.9)	96 (85.0)	<0.001		7834 (98.9)	225 (91.1)		<0.001	
	Yes	90 (1.1)	17 (15.0)			85 (1.1)	22 (8.9)		
Exposure (%)	No	7590 (94.3)	76 (67.3)	<0.001		7471 (94.3)	195 (78.9)		<0.001	
	Yes	463 (5.7)	37 (32.7)			448 (5.7)	52 (21.1)			

At the manufacturer recommended threshold, the phi correlation coefficient for the Abbott and EI qualitative results among females (Φ = 0.47) was 34% greater than males (Φ = 0.35). Increasing the EI threshold among females to 1.4 reduced EI positive discordance by 39% without reducing positive concordance. Among males, an adjustment in the EI threshold to 1.8 reduced the EI positive discordance by 64% with the loss of a single positive concordance result. Any reduction in the threshold had a negative impact on discordance among both sexes. Further, a decrease in correlation was observed among both sexes and subgroups when classifying EI borderline results as positive (Table 2).

**Table 2:** Phi correlation coefficients of Abbott and EI qualitative results. The table shows the phi correlation coefficients of the Abbott and EI qualitative results among (A) males (*n* = 3,801) and (B) females (*n* = 4,365) in the low prevalence subgroup at manufacturer-recommended and adjusted thresholds. The highlighted cell represents the manufacturer recommended cutpoints for Abbott and EI positive results, 1.4 and 1.1, respectively.

**Males**															
**EuroImmun Qualitative Threshold**															
Abbott Qualitative Threshold		**0.7**	**0.8**	**0.9**	**1.0**	**1.1**	**1.2**	**1.3**	**1.4**	**1.5**	**1.6**	**1.7**	**1.8**	**1.9**	**2.0**
	**1.0**	0.23	0.28	0.31	0.33	0.36	0.35	0.37	0.39	0.40	0.43	0.45	0.45	0.45	0.45
	**1.1**	0.22	0.26	0.29	0.32	0.35	0.35	0.37	0.39	0.41	0.44	0.45	0.45	0.45	0.45
	**1.2**	0.23	0.27	0.30	0.33	0.36	0.37	0.39	0.41	0.43	0.46	0.48	0.48	0.47	0.48
	**1.3**	0.24	0.28	0.31	0.33	0.36	0.37	0.40	0.42	0.43	0.46	0.48	0.50	0.49	0.49
	**1.4**	0.23	0.27	0.30	0.32	**0.35**	0.37	0.39	0.42	0.44	0.46	0.49	0.50	0.50	0.50
	**1.5**	0.23	0.27	0.30	0.35	0.36	0.37	0.39	0.43	0.44	0.47	0.49	0.51	0.50	0.51
	**1.6**	0.25	0.29	0.32	0.35	0.38	0.39	0.42	0.45	0.47	0.47	0.52	0.54	0.53	0.53
	**1.7**	0.25	0.29	0.32	0.35	0.38	0.39	0.42	0.45	0.47	0.50	0.52	0.54	0.53	0.53
	**1.8**	0.25	0.29	0.33	0.35	0.39	0.39	0.42	0.46	0.47	0.51	0.53	0.54	0.54	0.54

		***Females***													
		**EuroImmun Qualitative Threshold**													
Abbott Qualitative Threshold		**0.7**	**0.8**	**0.9**	**1.0**	**1.1**	**1.2**	**1.3**	**1.4**	**1.5**	**1.6**	**1.7**	**1.8**	**1.9**	**2.0**
	**1.0**	0.29	0.34	0.38	0.40	0.42	0.45	0.46	0.49	0.50	0.49	0.52	0.53	0.53	0.59
	**1.1**	0.30	0.35	0.39	0.41	0.43	0.46	0.47	0.50	0.51	0.50	0.53	0.54	0.55	0.60
	**1.2**	0.30	0.35	0.39	0.41	0.43	0.46	0.48	0.50	0.51	0.50	0.54	0.54	0.55	0.61
	**1.3**	0.32	0.37	0.41	0.43	0.46	0.49	0.50	0.53	0.54	0.53	0.57	0.57	0.58	0.64
	**1.4**	0.32	0.37	0.41	0.43	**0.47**	0.49	0.51	0.54	0.55	0.54	0.58	0.58	0.59	0.65
	**1.5**	0.32	0.37	0.42	0.45	0.49	0.52	0.54	0.56	0.58	0.56	0.60	0.61	0.61	0.68
	**1.6**	0.32	0.37	0.42	0.45	0.49	0.52	0.54	0.56	0.58	0.56	0.60	0.61	0.61	0.68
	**1.7**	0.33	0.38	0.43	0.46	0.50	0.53	0.55	0.58	0.59	0.58	0.62	0.63	0.63	0.70
	**1.8**	0.35	0.40	0.45	0.48	0.52	0.55	0.57	0.60	0.62	0.60	0.64	0.65	0.66	0.72

**Table 3:** Demographic and epidemiologic factors stratified by prevalence subgroup. The table shows the demographic and epidemiologic factors stratified by high and lowprevalence subgroups. Participants who self-reported a previous positive PCR, had a positive PCR at the time of screening, claimed anosmia or ageusia after March 1, 2020, or claimed an exposure with a known case were classified as the high-prevalence subgroup. The remaining participants were categorized in the low prevalence subgroup. Fisher’s exact test was used to evaluate null and alternate hypothesis. H_0_: the prevalence subgroup and parameters are independent. H_1_: the prevalence subgroup and parameters are not independent.

	**Level**	**High prevalence subgroup**	**Low prevalence subgroup**	*p*
N		611	7,555	
Sex (%)	Female	320 (52.4)	4,045 (53.5)	0.607
	Male	291 (47.6)	3,510 (46.5)	
Age (mean (SD))		41.9 (16.4)	45.1 (19.1)	<0.001
Race (%)	American Indian or Alaska Native	6 (1.0)	39 (0.5)	<0.001
	Asian	12 (2.0)	182 (2.4)	
	Black	6 (1.0)	32 (0.4)	
	Multi-racial	24 (3.9)	154 (2.0)	
	Native Hawaiian or Other Pacific Islander	5 (0.8)	25 (0.3)	
	Unknown	32 (5.2)	174 (2.3)	
	White	526 (86.1)	6,949 (92.0)	
Ethnicity (%)	Hispanic	92 (15.1)	636 (8.4)	<0.001
	Non-Hispanic	519 (84.9)	6,919 (91.6)	
Diabetes (%)	No	580 (94.9)	7,058 (93.4)	0.171
	Yes	31 (5.1)	497 (6.6)	
Cardiovascular Disease (%)	No	593 (97.1)	7,230 (95.7)	0.133
	Yes	18 (2.9)	325 (4.3)	
Hypertension (%)	No	543 (88.9)	6,546 (86.6)	0.133
	Yes	68 (11.1)	1,009 (13.4)	
Immunosuppressive Therapy (%)	No	604 (98.9)	7,482 (99.0)	0.826
	Yes	7 (1.1)	73 (1.0)	
Autoimmune Disease (%)	No	574 (93.9)	7,166 (94.9)	0.382
	Yes	37 (6.1)	389 (5.1)	
Concordance (%)	Abbott Positive Discordance	6 (1.0)	38 (0.5)	<0.001
	EI Positive Discordance	26 (4.3)	152 (2.0)	
	Negative Concordance	527 (86.3)	7,348 (97.3)	
	Positive Concordance	52 (8.5)	17 (0.2)	

### Low and high prevalence subgroups

Of the 8,166 in the study population, 92.5% (*n* = 7,555) were in the low prevalence subgroup as defined above. The EI positivity was higher than Abbott’s positivity in both the high prevalence (EI: *n* = 78 (12.8%), Abbott: *n* = 33 (9.5%) and low prevalence (EI: *n* = 169 (2.2%), Abbott: *n* = 22 (0.7%)) subgroups. Of 84 individuals in the high prevalence subgroup with at least one of the two serology assays testing positive, 52 (61.9%) were positive in both tests. In contrast, only 17 of 207 (8.2%) individuals in the low prevalence subgroup with at least one positive serology assay tested positive for both. Several differences in demographics were observed between low- and high-prevalence subgroups, including a higher mean age, a greater proportion of white participants, and fewer Hispanic participants in the low prevalence subgroup. No significant differences in sex or comorbidities were observed between the subgroups (Table 3).

**Table 4:** Demographic and epidemiologic factors stratified by sex in the low prevalence subgroup. The table shows demographic and epidemiologic factors stratified by sex in the low-prevalence subgroup. The low prevalence subgroup was comprised of participants claiming no previous positive PCR, negative PCR at the time of screening, no history of anosmia or ageusia after March 1, 2020, no known exposures. Fisher’s exact test was used to evaluate null and alternate hypothesis. H_0_: sex and parameters are independent. H_1_: sex result and parameters are not independent.

	**Level**	**Female**	**Male**	*p*
N		4,045	3,510	
Age (mean (SD))		45.0 (18.8)	45.07 (19.4)	0.960
Race (%)	Unknown	103 (2.5)	71 (2.0)	0.099
	American Indian or Alaska Native	23 (0.6)	16 (0.5)	
	Asian	112 (2.8)	70 (2.0)	
	Black	14 (0.3)	18 (0.5)	
	Multi-racial	82 (2.0)	72 (2.1)	
	Native Hawaiian or Other Pacific Islander	10 (0.2)	15 (0.4)	
	White	3701 (91.5)	3248 (92.5)	
Ethnicity (%)	Hispanic	374 (9.2)	262 (7.5)	0.006
	Non-Hispanic	3671 (90.8)	3248 (92.5)	
Diabetes (%)	No	3804 (94.0)	3254 (92.7)	0.022
	Yes	241 (6.0)	256 (7.3)	
Cardiovascular Disease (%)	No	3929 (97.1)	3301 (94.0)	<0.001
	Yes	116 (2.9)	209 (6.0)	
Hypertension (%)	No	3565 (88.1)	2981 (84.9)	<0.001
	Yes	480 (11.9)	529 (15.1)	
Immunosuppressive Therapy (%)	No	3994 (98.7)	3488 (99.4)	0.007
	Yes	51 (1.3)	22 (0.6)	
Autoimmune Disease (%)	No	3746 (92.6)	3420 (97.4)	<0.001
	Yes	299 (7.4)	90 (2.6)	
Concordance (%)	Abbott Positive Discordance	23 (0.6)	15 (0.4)	<0.001
	EI Positive Discordance	48 (1.2)	104 (3.0)	
	Negative Concordance	3964 (98.0)	3384 (96.4)	
	Positive Concordance	10 (0.2)	7 (0.2)	

To focus on false positives, the analysis was then restricted to the low prevalence subgroup. Males comprised 46.5% of this subgroup but contributed to 104 (68.4%) of 152 EI positive discordant results. Additionally, a greater proportion of males claimed diabetes (7.3%) cardiovascular disease (6.0%) and hypertension (15.1%) than females (6.0%, 2.9%, and 11.9%, respectively). A higher proportion of females reported immunosuppressive therapy (1.3%) and autoimmune disease (7.4%) than males (0.6% and 2.6%, respectively). A greater fraction of females in the low prevalence subgroup self-reported Hispanic ethnicity than males. No significant differences between sexes were observed for age or race (Table 4).

**Table 5:** Association between males and assay results in the low prevalence subgroup. The table shows the association (OR and 95% CI) between males and positive EI and Abbott results in the low prevalence subgroup. The fully adjusted model included age, race, ethnicity, diabetes, cardiovascular disease, hypertension, immunosuppressive therapy, autoimmune disease, and household.

		**Crude**		**Adjusted**	
		**OR**		**95% CI**	**OR**	**95% CI**
**EuroImmun**				
	Male	2.24		1.63–3.09	3.12	2.04–4.78
**Abbott**				
	Male	0.77		0.45–1.32	0.80	0.41–1.58

Summary statistics for the high prevalence subgroup stratified by sex can be found in the (Table S1).

In the low prevalence subgroup, males were significantly associated with a positive EI result (OR: 2.24, CI: 1.63, 3.09). The model—which adjusted for age, race, ethnicity, diabetes, cardiovascular disease, hypertension, immunosuppressive therapy, autoimmune disease, and household clustering—yielded an even stronger association between males and positive EI result (adjusted OR: 3.12, CI: 2.04, 4.78) (Table 5). Additionally, autoimmune disease was significantly associated with EI positivity (OR: 3.30, CI: 1.42, 7.67). No other factors in the model were associated with an EI positive result.

Sensitivity analyses were performed at the alternate EI thresholds for positivity (1.4 and 1.8) yielded similar results (OR: 3.01 and 2.95, respectively).

A similar analysis of Abbott positivity in the low prevalence subgroup did not find an association with any of the covariates examined. In particular, race and ethnicity were no longer associated with a positive Abbott test, as observed in the crude results (Table 1).

Neither assay was significantly associated with any covariates examined in crude or adjusted models in the high prevalence subgroup.

## Discussion

This study evaluated the side-by-side performance of Abbott and EI in a seroprevalence survey. A significant qualitative discordance was observed between Abbott and EI. While several factors were identified to be strongly associated with this discordance, a significant sex imbalance was identified among EI positives that was not present among Abbott positives. Discordance between the evaluated assays is well described in the literature; however, the identification of sex-driven false positivity of EI relative to Abbott provides potential understanding of factors contributing to the described discordance.

As the focus of this investigation was to evaluate the issue of false positivity, we capitalized on the low prevalence subgroup, in which the proportion of false positives are likely to be higher, to evaluate factors contributing to discordant results. Overall, EI was associated with more false positives than Abbott, with a substantial imbalance between sexes. This association was isolated to EI and not evident with Abbott. While studies have identified a rapid decline in anti-N following infection among males (Grzelak et al., 2021), our choice to restrict our analyses to the low prevalence subgroup reduced the potential of this decline to impact the results. Further, as sex was not significantly associated with an EI-positive result in the high prevalence subgroup, our findings suggest a reduced positive predictive value for the EI assay in low prevalence populations.

Given the sex-centric discordance observed between assays, alternative sex-specific thresholds for a positive result were evaluated to determine the implications on false positivity. At the Abbott and EI recommended thresholds, females were associated with greater concordance than males. While the observed discordance among males was greater than females at all observed thresholds, a significant improvement in sex-specific concordance as the EI threshold was increased was observed with a minimal reduction in the frequency of positive concordant results. Sensitivity analyses of alternate threshold for EI positivity, (cut point: 1.4 and 1.8), produced similar results, likely due to a reduction in both male and female discordance. Reductions below the EI recommended cut-point for positivity were associated with significant reductions in concordance. These results are consistent with those in previous literature, which described that the classification of indeterminate results (quantitative value of 0.08−1.0) as positive negatively impacts the assay’s specificity (Prince et al., 2020; Stocking et al., 2022; Tang et al., 2020). These results suggest the need to evaluate the benefit of sex-specific positivity threshold to improve the performance of EI.

Seroprevalence studies are an essential tool for public health responses against infectious diseases and provide information for estimating transmission intensity and population susceptibility (Bryant et al., 2020). In the COVID-19 pandemic alone, thousands of seroprevalence studies have been conducted, with many used to provide data on the burden of infection in their respective regions (Chen et al., 2021b). An accurate seroprevalence estimate includes a correction for test performance, which is almost exclusively reported in a sex-independent manner. Considering these findings, it is important to evaluate sex-specific corrections for test performance in seroprevalence estimates.

Our results are consistent with the literature, which has described a variety of specificity limitations with EI (Prince et al., 2020; Tang et al., 2020). This includes EI’s Information for Use which suggests potential cross-reactivity with several commonly circulating respiratory pathogens, including respiratory syncytial virus. While we were unable to control for cross-reactivity in this analysis, Nawrocki et al. (2021) identified a dramatic decline in several of these pathogens early in the pandemic due to non-pharmaceutical interventions aimed at reducing SARS-CoV-2. This nationwide reduction in pathogens of potential cross-reactivity during the study period and the magnitude of the discordance suggest the observed association is unlikely to be due to cross-reactivity alone. However, this impact should be reevaluated as common respiratory pathogens return to pre-pandemic levels.

This evaluation was limited to the data collected as a part of the HERO Project in Utah. Due to the racial homogeneity of the population, further investigations are necessary to evaluate if the findings hold for additional races.

Lastly, investigators had limited resolution into true positivity or time since positivity, limiting the ability to assess sensitivity in the low prevalence subgroup. Further evaluation of sex-specific sensitivity and specificity of anti-N and anti-S assays is necessary to provide insight into the potential limitations and benefits of sex-specific thresholds for EI and evaluate the generalizability to other anti-S assays. Further, a better understanding of the effect of sex on the performance of these assays is critical in the post-vaccination era to effectively measure the population’s immune response to the vaccine and previous infections.

## Conclusions

Significant quantitative and qualitative discordance was observed between Abbott and EI throughout the seroprevalence study. Higher positivity was observed in EI than among Abbot. Investigators observed disproportionately positive EI results among men; however, this was not observed with Abbott. The association between the male sex and EI seropositivity was particularly strong in the low-prevalence subgroup. Our results suggest there may be significant sex-associated specificity limitations with the EI assay. As these findings may extend to other anti-S assays utilized for SARS-CoV-2 seroprevalence investigations, further investigation is needed to evaluate the generalizability of these findings with other manufactures’ assays.

##  Supplemental Information

10.7717/peerj.15247/supp-1 Supplemental Information 1 Data for participants that were evaluated by Abbott and EI assays as part of the HERO Project between June 10 to August 15, 2020 and completed the associated survey

10.7717/peerj.15247/supp-2 Supplemental Information 2 Demographic and Epidemiologic Factors Stratified by Sex in the High-Prevalence Subgroup The low prevalence subgroup was comprised of participants claiming no previous positive PCR, negative PCR at the time of screening, no history of anosmia or ageusia after March 1, 2020, no known exposures. Fishers exact test was used to evaluate null and alternate hypothesis. *H*_0_: sex and parameters are independent. *H*_1_: sex and parameters are not independent.

## Additional Information and Declarations

### Data Availability

The following information was supplied regarding data availability: The dataset is available in the Supplementary File.
