# Supplementary material for: Sex difference in the discordance between Abbott Architect and EuroImmun serological assays
Source: PeerJ. 2023 Jul 17;11:e15247. doi: 10.7717/peerj.15247 (PMC10358334; doi:10.7717/peerj.15247)
Supplement: Supplemental Information 2 — The low prevalence subgroup was comprised of participants claiming no previous positive PCR, negative PCR at the time of screening, no history of anosmia or ageusia after March 1, 2020, no known exposures. Fishers exact test was used to evaluate null and alternate hypothesis. H0: sex and parameters are independent. H1: sex and parameters are not independent. [file peerj-11-15247-s002.docx]

|  | **Level** | **Female** | **Male** | **p** |
| --- | --- | --- | --- | --- |
| N |  | 320 | 291 |  |
| Age (mean (SD)) |  | 42.43 (16.36) | 41.40 (16.47) | 0.437 |
| Race (%) | Unknown | 20 ( 6.2) | 12 ( 4.1) | 0.108 |
|  | American Indian or Alaska Native | 1 ( 0.3) | 5 ( 1.7) |  |
|  | Asian | 8 ( 2.5) | 4 ( 1.4) |  |
|  | Black | 3 ( 0.9) | 3 ( 1.0) |  |
|  | Multi-racial | 11 ( 3.4) | 13 ( 4.5) |  |
|  | Native Hawaiian or Other Pacific Islander | 5 ( 1.6) | 0 ( 0.0) |  |
|  | White | 272 ( 85.0) | 254 ( 87.3) |  |
| Ethnicity (%) | Hispanic | 47 ( 14.7) | 45 ( 15.5) | 0.877 |
|  | Non-Hispanic | 273 ( 85.3) | 246 ( 84.5) |  |
| Diabetes (%) | No | 304 ( 95.0) | 276 ( 94.8) | 1 |
|  | Yes | 16 ( 5.0) | 15 ( 5.2) |  |
| Cardiovascular Disease (%) | No | 316 ( 98.8) | 277 ( 95.2) | 0.018 |
|  | Yes | 4 ( 1.2) | 14 ( 4.8) |  |
| Hypertension (%) | No | 289 ( 90.3) | 254 ( 87.3) | 0.289 |
|  | Yes | 31 ( 9.7) | 37 ( 12.7) |  |
| Immunosuppressive Therapy (%) | No | 316 ( 98.8) | 288 ( 99.0) | 1 |
|  | Yes | 4 ( 1.2) | 3 ( 1.0) |  |
| Autoimmune Disease (%) | No | 297 ( 92.8) | 277 ( 95.2) | 0.289 |
|  | Yes | 23 ( 7.2) | 14 ( 4.8) |  |
| Concordance (%) | Abbott Positive Discordance | 3 ( 0.9) | 3 ( 1.0) | 0.767 |
|  | EI Positive Discordance | 11 ( 3.4) | 15 ( 5.2) |  |
|  | Negative Concordance | 279 ( 87.2) | 248 ( 85.2) |  |
|  | Positive Concordance | 27 ( 8.4) | 25 ( 8.6) |  |
